# A single cation or anion dendrimer-based liquid electrolyte†
†Electronic supplementary information (ESI) available. See DOI: 10.1039/c5sc04584c


**DOI:** 10.1039/c5sc04584c

**Published:** 2016-01-29

**Authors:** Sudeshna Sen, Rudresha B. Jayappa, Haijin Zhu, Maria Forsyth, Aninda J. Bhattacharyya

**Affiliations:** a Solid State and Structural Chemistry Unit , Indian Institute of Science , Bangalore , 560012 , India . Email: aninda_jb@sscu.iisc.ernet.in; b Institute for Frontier Materials , Deakin University , Burwood , Waurn Ponds , VIC3216 , Australia

## Abstract

We propose here a novel liquid dendrimer-based single ion conductor as a potential alternative to conventional molecular liquid solvent–salt solutions in rechargeable batteries, sensors and actuators. A specific change from ester (–COOR) to cyano (–CN) terminated peripheral groups in generation-one poly(propyl ether imine) (G_1_-PETIM)–lithium salt complexes results in a remarkable switchover from a high cation (*t*_Li^+^_ = 0.9 for –COOR) to a high anion (*t*_PF_6_^–^_ = 0.8 for –CN) transference number. This observed switchover draws an interesting analogy with the concept of heterogeneous doping, applied successfully to account for similar changes in ionic conductivity arising out of dispersion of insulator particle inclusions in weak inorganic solid electrolytes. The change in peripheral group simultaneously affects the effective ionic conductivity, with the room temperature ionic conductivity of PETIM–CN (1.9 × 10^–5^ Ω^–1^ cm^–1^) being an order of magnitude higher than PETIM–COOR (1.9 × 10^–6^ Ω^–1^ cm^–1^). Notably, no significant changes are observed in the lithium mobility even following changes in viscosity due to the change in the peripheral group. Changes in the peripheral chemical functionality directly influence the anion mobility, being lower in PETIM–COOR than in PETIM–CN, which ultimately becomes the sole parameter controlling the effective transport and electrochemical properties of the dendrimer electrolytes.

## Abstract 2

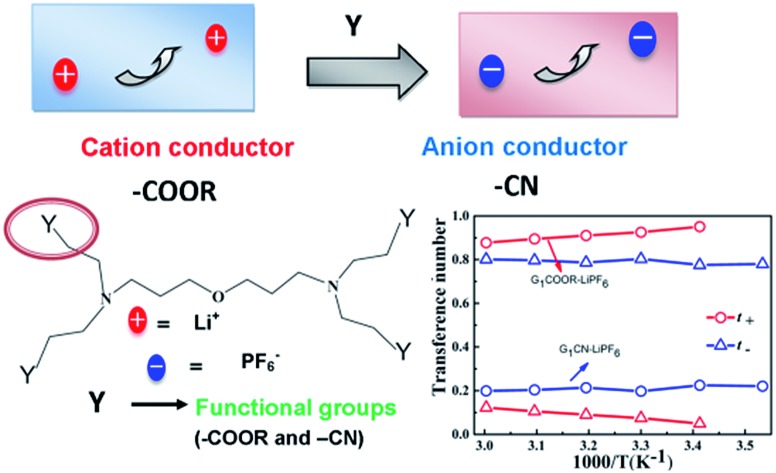
The proposed dendrimer based liquid electrolyte is a single-ion conductor where ion transport is altered by the nature of the chemical functionalities leading to large variations in anion diffusion and hence ionic transference number.

## Introduction

One of the important strategies towards building stable and safe rechargeable batteries has been to develop newer forms of electrolytes as potential alternatives to conventional liquid molecular solvent–salt solutions.^1^,^2^ In this line of thought, various electrolyte systems ranging from solid crystalline electrolytes to “solid-like” soft organic electrolytes have been explored as alternatives to conventional liquid electrolytes focused mainly for applications in lithium-based battery chemistries.^3^–^7^ Polymer electrolytes, which exhibit interesting compliable mechanical properties in addition to high ionic conductivity, have shown greater potential than solid crystalline electrolytes in diverse electrochemical devices, *viz.* batteries,8*a* fuel cells,8*b* actuators,8*c* sensors.^9^ Design of novel polymer architectures (*e.g.* network, branched polymers) has been one of the important strategies for the development of high ion conducting polymer electrolytes.^10^ The major drawback with various polymer-based electrolytes is that the cations and anions contribute to the specific conductivity and thus the specific ion, *i.e.* cation or anion, transference number is not high. In particular the cation ion transference number, which is of practical interest for various rechargeable battery chemistries, is typically low in the range *t*_+_ = 0.2–0.5.^10^,^11^ There have been several strategies to design polymer electrolytes with high transference number without significantly compromising the effective ionic conductivity. The majority of these approaches have been applied on polymers in the solid form, *viz. via* chemical manipulations of the constituting units of the polymer or from single ion conductors where an ion of one type (say anion) is immobilized on the polymer backbone as in block or copolymer units. The other major concern with solid-like electrolytes is related to poor charge transport kinetics at the electrode|electrolyte interface which leads to poor device efficiency. Apart from a few glowing examples, the overwhelming majority of polymer electrolytes and in general solid electrolytes have not been able to successfully transcend the precincts of laboratory-scale demonstrations. This has led to the persistence of conventional liquid electrolytes in the majority of modern-day electrochemical devices, including rechargeable batteries.

Dendrimers are a special class of mono-dispersed branched polymers, containing a large number of branched flexible chain-ends emanating from a core or linker molecules. This unique architecture has attracted considerable attention in biomedicine, catalysis, sensing and energy storage.^12^ The mechanical consistency of dendrimers is intermediate between low viscosity molecular solvents (*η* ≈ 10^–3^ Pa s) and high viscosity polymer gel or polymer–salt complex (very high *η*; *η* → ∞) electrolytes. Due to the higher viscosities of dendrimers compared to typical molecular solvents, the ionic mobility and hence the effective ionic conductivity of dendrimer electrolytes are expected to be lower compared to molecular solvent–salt liquid solutions. In the context of the vast volume of work accomplished with regard to solid polymers with high ionic conductivity,^13^ viscosity cannot be the sole criteria for the determination of ionic mobility and effective ionic conductivity. Similarly, the high viscosity of dendrimers should not be a deterrent for exploring their application in electrochemical devices. The high degree of branching in the dendrimer network leads to multiple advantages, *viz.* larger free volume, higher amorphicity and low glass transition temperatures (implying higher chain flexibility).^14^ These, coupled with the flexibility to freely tune the chemical composition and conformation as a function of generation number, also significantly affect the ion-solvating ability and host–guest interactions with various metal salts including alkali-metal salts which are of direct relevance to rechargeable batteries. These advantageous features should make dendrimers an attractive alternative liquid matrix to conventional liquids, ionic liquids or solid polymers for the synthesis of ion conductors tailored to perform specific tasks in various electrochemical devices. Of specific interest is whether a dendrimer can be employed to produce electrolytes with high ion transference number of a single ion type. To the best of our knowledge there have been no efforts undertaken in this direction. Additionally, there have been no detailed and conclusive studies undertaken on the correlation of various chemical functional parameters with the ion transport mechanism in dendrimers, in spite of their anticipated potential in various electrochemical applications. Studies in these directions will be expected to throw more light on the ion transport mechanism in dendrimers and identify key parameters for the development of dendrimer-based ion conductors for various applications such as rechargeable batteries, sensors, and actuators.^14^,^15^ We present here for the first time a detailed study of the influence of the chemical nature of peripheral functional groups on ionic conductivity, diffusion and transference number in generation-1 poly(propyl ether imine) (G_1_-PETIM)–lithium salt mixtures. We demonstrate here that the peripheral chemical functionality is a very important parameter to optimize the effective transport as well as the electrochemical properties of the dendrimer electrolyte.

## Results and discussion

**Fig. 1:**
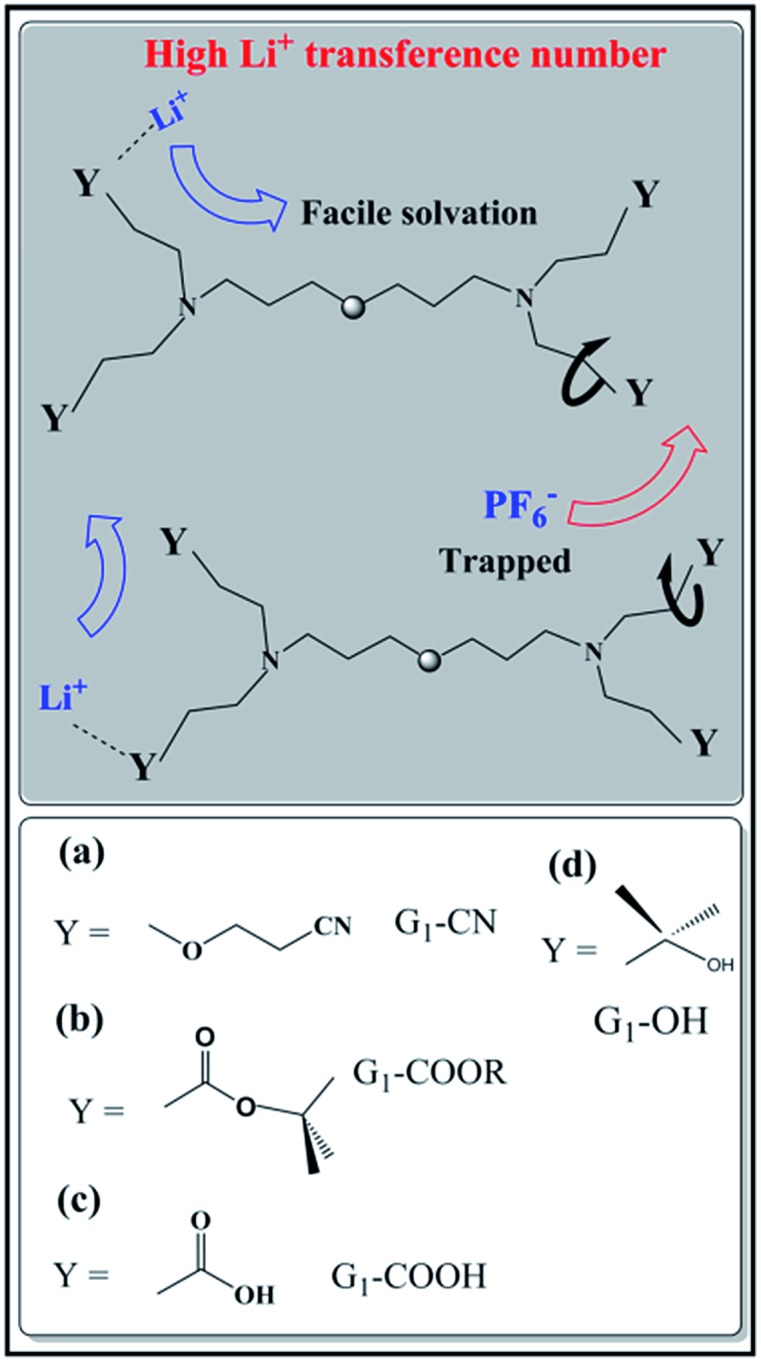
Schematic representation of ion transport mechanism in G_1_–PETIM dendrimers with different peripheral functional groups: (a) G_1_–CN, (b) G_1_–COOR, (c) G_1_–COOH, (d) G_1_–OH.

Ion transport in polymer electrolytes largely depends on the chemical characteristics of the polymer, such as branching, network, and functionality of the side chains.8a,^16^,^17^ Different functional groups exhibit different binding abilities due to varying polarity of the groups and this influences the ion solvation *via* dissociation of the salt. On the other hand, their spatial distributions and the sizes of end chains affect both cation and anion mobility, resulting in large differences in the corresponding ion transference numbers.^17^ The above stated issues on ionic conductivity and transference number are systematically probed here in the context of G_1_-PETIM dendrimers. The G_1_-dendrimers are synthesized with different end functional groups (–COOH, –COOR, –OH, –CN) maintaining the same linker (ether) and branching points (tertiary amine; *cf.* schematic Fig. 1). The study here focuses only on the first generation dendrimers primarily due to the following reasons. Firstly, the viscosity (0.1–6 Pa s) of the G_1_-PETIM dendrimer, though higher than the viscosity of a typical liquid molecular solvent (0.5–10 mPa s),^18^ is much lower than the higher generation G_*n*_-PETIM dendrimers (*n* = 2–4). So, the influence of viscosity on ionic conductivity will be much less in G_1_-PETIM compared to G_*n*_-PETIM dendrimer liquid electrolytes. The magnitude of ionic conductivity of some of the G_1_ dendrimers will not be significantly lower compared to a typical molecular liquid solvent–salt solution^17^ of relevance to rechargeable batteries (typically: >10^–3^ to 10^–2^ Ω^–1^ cm^–1^). Secondly, in G_1_ dendrimers the number density of the linker and branching moieties is lower compared to higher generation dendrimers. Hence, the non-trivial influence due to the linker and branching moieties on solvation and ion mobility anticipated in the higher generations will be minimal and assumed constant in the G_1_ dendrimers. The ion transport in the G_1_ dendrimer electrolytes can then be directly correlated to the nature of the peripheral chemical functionality.

**Fig. 2:**
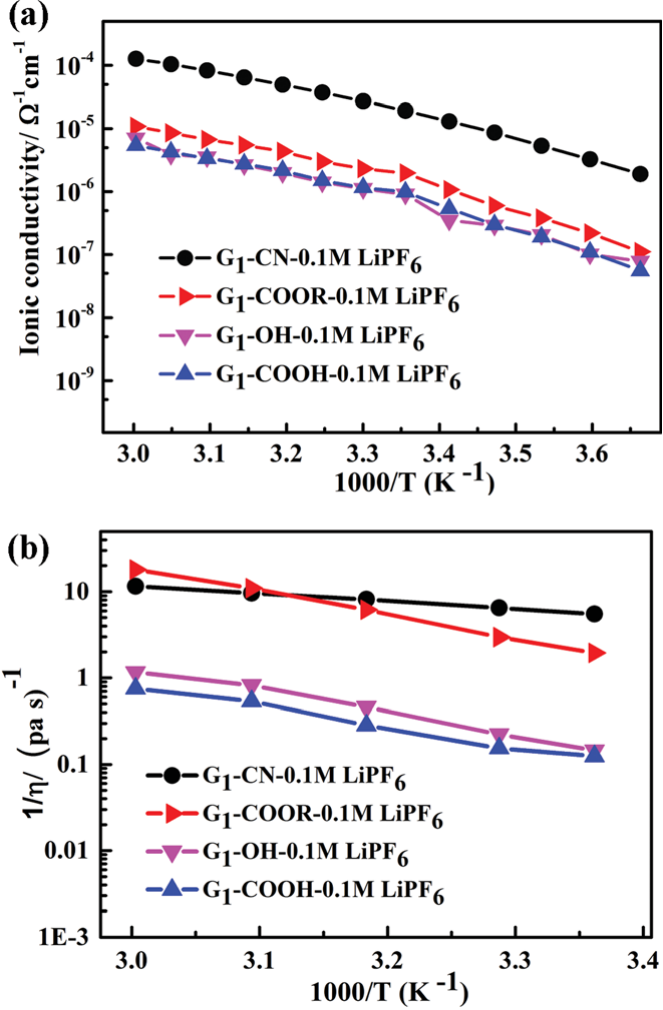
(a) Temperature dependent ionic conductivity and (b) fluidity (*η*^–1^) of G_1_ dendrimers with different peripheral functional groups.

The temperature dependent ionic conductivity and viscosity of first-generation dendrimers (with different functional groups) with 0.1 M LiPF_6_ in the temperature range 0–60 °C are shown in Fig. 2a and b respectively. The first-generation nitrile-terminated PETIM dendrimer electrolyte (G_1_–CN–0.1 M LiPF_6_) exhibits the highest room temperature ionic conductivity of 1.9 × 10^–5^ Ω^–1^ cm^–1^, whereas G_1_–OH–0.1 M LiPF_6_ shows the lowest conductivity of 9 × 10^–7^ Ω^–1^ cm^–1^. The conductivities of G_1_–COOR–0.1 M LiPF_6_ and G_1_–COOH–0.1 M LiPF_6_ at 25 °C are intermediate to those of G_1_–CN–0.1 M LiPF_6_ and G_1_–OH–LiPF_6_, being 1.9 × 10^–6^ Ω^–1^ cm^–1^ and 9.8 × 10^–7^ Ω^–1^ cm^–1^ respectively. The measured ionic conductivity values of the various dendrimer electrolytes, which are on a par with many single ion conductors,10a,^19^ have the following trend: *σ*_G_1_–CN_ > *σ*_G_1_–COOR_ > *σ*_G_1_–COOH_ ∼ *σ*_G_1_–OH_. This variation in conductivity between the various dendrimer electrolytes by more than one order of magnitude indicates a strong correlation between the chemical nature of peripheral functionalization and ion transport. The activation energies of conductivity are obtained by a linear fit of the conductivity data using the Arrhenius equation: *σ* = *A*e^–*E*_a_/*kT*^, where *A*, *E*_a_, *k*, *T* are the pre-exponential factor, activation energy, Boltzmann constant and temperature respectively (*cf.* fitting parameters in Table ST2†). In spite of notable differences in ionic conductivities between G_1_–CN, G_1_–COOR and G_1_–COOH, interestingly no significant differences exist in the estimated activation energies between G_1_–CN (*Eσ*_G_1_–CN_ = 0.54 eV), G_1_–COOR (*Eσ*_G_1–_COOR_ = 0.58 eV) and G_1_–COOH (*Eσ*_G_1–_COOH_ = 0.58 eV). The VTF fitting^20^ parameters of the conductivity plot (Fig. 2a) are tabulated in Table ST3.† However, it is strongly felt that VTF fitting of the conductivity results is inappropriate for the present study. This is mainly attributed to the simpler chemical structure of the first-generation dendrimers compared to higher generation dendrimers and polymers.

The conductivity behaviour is correlated to the temperature dependent fluidity (1/*η*, where *η* is the viscosity) shown in Fig. 2b. The viscosities of PETIM dendrimers, calculated from the static viscosity *versus* shear rate measurements, are shown in Table ST2.† The viscosity increases by nearly two times from 0.15 Pa s for G_1_–CN to 0.30 Pa s for G_1_–COOR. The viscosities at 30 °C for G_1_–OH–0.10 M LiPF_6_ and G_1_–COOH–0.10 M LiPF_6_ are even higher, being 4.5 Pa s and 6.5 Pa s respectively. The viscosity values showed the following trend: *η*_G_1_–CN_ < *η*_G_1_–COOR_ < *η*_G_1_–OH_ < *η*_G_1_–COOH_. The higher viscosity of the ester dendrimer (G_1_–COOR) compared to G_1_–CN is attributed to the more polar nature of the –COOR group compared to the –CN group and the steric hindrance exerted by the bulkier –COOR (R = *t*-butyl) group implying a higher dragging force compared to the linear –CN in G_1_–CN. Differences in viscosity between G_1_–carboxyl/hydroxyl groups (*i.e.* COOR, COOH and OH) and G_1_–CN can also be accounted for on the basis of the intra- or inter-molecular hydrogen bonding. The strength of hydrogen bonds is significantly higher in G_1_–COOH and G_1_–OH resulting in significantly higher viscosity compared to G_1_–CN and G_1_–COOR. An increase in viscosity for carboxyl groups (G_1_–COOR and G_1_–COOH) and hydroxyl (G_1_–OH) terminated dendrimers results in a decrease in ionic conductivity compared to the G_1_–CN dendrimer. Activation energies of viscosity, obtained by fitting the temperature dependent fluidity (1/*η*) (Fig. 2b) using the Arrhenius equation, are tabulated in Table ST2.† Similar trends in activation energies for both temperature dependent conductivity and viscosity indicate that the underlying mechanism for conductivity and viscosity is thermally activated. However, the observed trends in viscosity cannot be correlated one-to-one with the conductivity trends. This is especially true for the –COOH terminated dendrimer. A logical interpretation of the observed trends in conductivity and viscosity can only be achieved by simultaneously studying the diffusion behavior of the various participating entities, *viz.* Li^+^, PF_6_^–^ and H (dendrimer). G_1_–COOH and G_1_–OH have been excluded from additional studies as both have lower ionic conductivities and higher viscosities compared to G_1_–CN and G_1_–COOR in the measured temperature range. G_1_–COOR is selected as the representative among the two carboxyl groups as an understanding of the mechanism in –COOR will also aid in accounting for the experimental observations in –COOH.

**Fig. 3:**
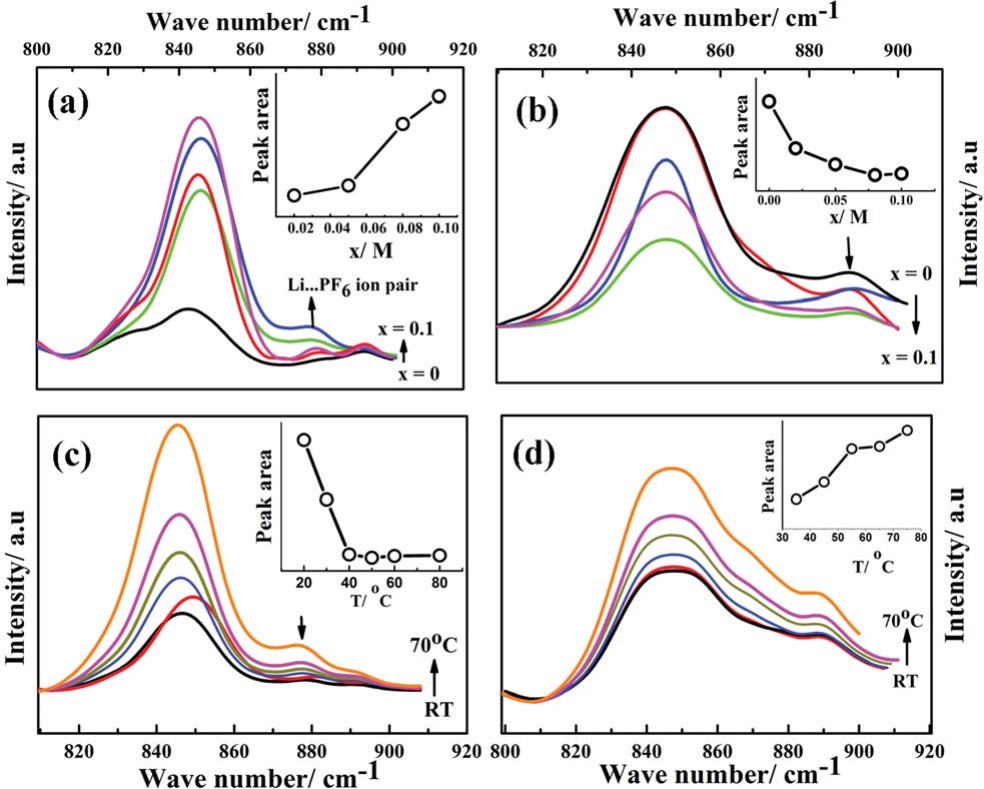
FTIR spectra of G_1_–CN–LiPF_6_ (a) and G_1_–COOR–LiPF_6_ (b) at different salt concentrations [*x* = 0 (black), 0.01 (red), 0.02 (magenta), 0.05 (green), 0.1 (blue)]. FTIR spectra of G_1_–CN–LiPF_6_ (c) and G_1_–COOR–LiPF_6_ (d) at different temperatures (*T* = RT to 70 °C, with *x* = 0.1) [*T* = RT (black), 30 °C (red), 40 °C (blue), 50 °C (green), 60 °C (magenta), 70 °C (orange)].

Ion solvation in G_1_–CN–LiPF_6_ and G_1_–COOR–LiPF_6_ electrolytes was studied by FTIR spectroscopy at various salt concentrations (ranging from 0 to 0.1 M) and temperatures (from room temperature to 70 °C). The FTIR spectra for both the electrolytes in the wavenumber region 800–920 cm^–1^ (normalized with respect to the highest intensity peak at 1118 cm^–1^ for G_1_–CN and 1154 cm^–1^ for G_1_–COOR) are shown in Fig. 3. The area under the peak is calculated by fitting the spectra with Gaussian function. Fig. 3a and b depicts the FTIR spectra of G_1_–CN–*x* M LiPF_6_ and G_1_–COOR–*x* M LiPF_6_ respectively with varying salt concentration (*x*) ranging from 0 to 0.10 M. Both pristine G_1_–COOR and G_1_–CN exhibit the characteristic symmetric stretching vibration of C–O–C groups of the aliphatic ether present in the dendrimer core at 848 cm^–1^ (splitting in the C–O–C symmetric stretch in G_1_–CN leads to an additional band at 830 cm^–1^, possibly due to the presence of two types of ether groups in the core and at the periphery).^21^

This band is observed to merge with the strong band also appearing at 848 cm^–1^ corresponding to the P–F vibration of the free PF_6_^–^ ion of the salt^22^ in G_1_–CN–*x* M LiPF_6_. A weak band appearing at 875 cm^–1^ for G_1_–CN–*x* M LiPF_6_ is assigned to the associated tri-dentate ion pair of the LiPF_6_ salt and this is in good agreement with previous reports.^22^ With increasing salt concentration, the band area of the weaker band at 875 cm^–1^ is observed to intensify (inset of Fig. 3a) with respect to the stronger band (at 848 cm^–1^) for G_1_–CN–*x* M LiPF_6_. This suggests the presence of ion pairs in G_1_–CN–*x* M LiPF_6_. The intensity of this shoulder band at 875 cm^–1^ decreases with increasing temperature from RT to 70 °C (Fig. 3c), signifying the dissociation of ion pairs with increasing temperature. In the case of pure G_1_–COOR, the observed IR band at 898 cm^–1^ (Fig. 3b) is attributed to the C–C stretching frequencies of ester (O–C–C) or ether groups,^21^ which is affected by addition of the salt as well as temperature. No additional bands corresponding to ion pairs are observed in G_1_–COOR–*x* M LiPF_6_ (Fig. 3b) at various salt concentrations, signifying facile salt dissociation in ester functionalized dendrimers. Stronger interaction of the oxygen atom of the ester group with the Li^+^ ion, compared to the –CN group, leads to higher dissociation of ion pairs in G_1_–COOR. To support the FTIR observations, the Stokes equations (*cf.* ESI†) for both dendrimers have been investigated (Fig. S3†). Fig. S3† shows the product of dc conductivity (related to the number of free charges from the Stokes equation) and viscosity at various temperatures for both dendrimers. The constancy of this product (*i.e. Nq*^2^/6π*r*_s_ in eqn (SE1) in ESI†) at various temperatures for G_1_–COOR signifies no change in free charges with an increase of temperature, which is true for electrolytes with a fully dissociated salt. In the case of G_1_–CN, an increase in free charges is observed with an increase in temperature (in Fig. S3 in ESI†), which is attributed to an increase in salt dissociation with increasing temperature.

**Fig. 4:**
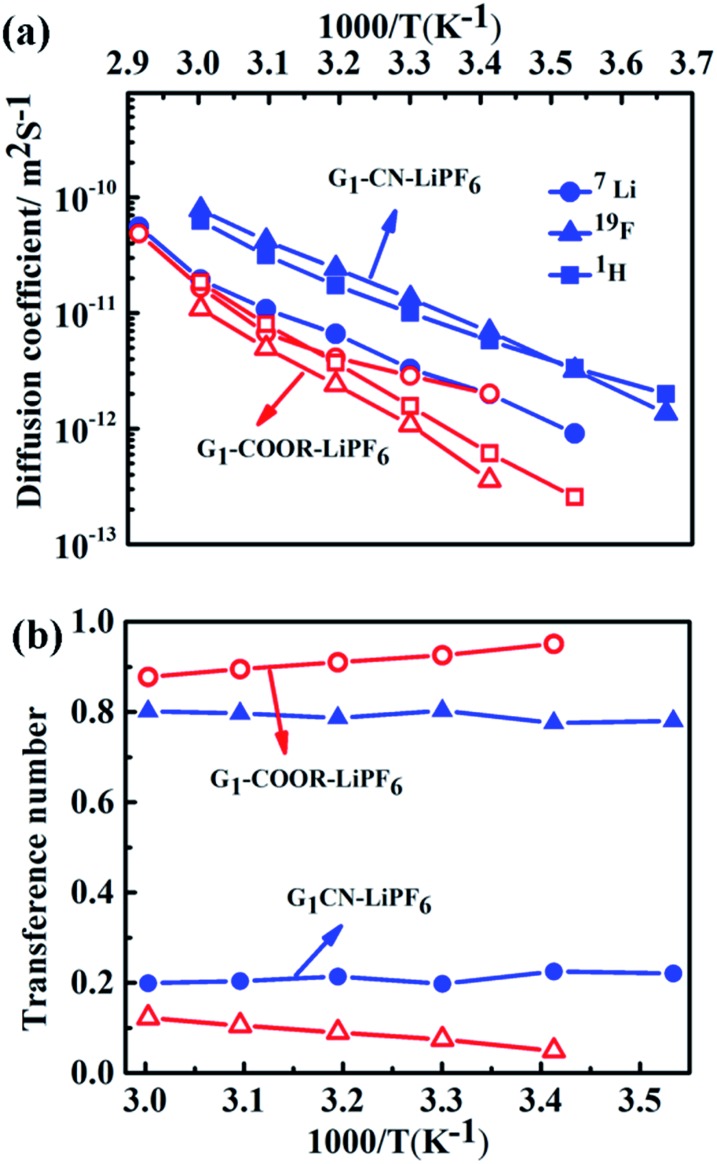
(a) Self-diffusion coefficients of ^1^H (squares), ^19^F (triangles), ^7^Li (circles) at various temperatures for G_1_–CN–0.1 M LiPF_6_ (blue, with closed symbols) and G_1_–COOR–0.1 M LiPF_6_ (red, with open symbols). (b) ^7^Li and ^19^F transference numbers of G_1_–CN–0.1 M LiPF_6_ (blue) and G_1_–COOR–0.1 M LiPF_6_ (red) at various temperatures.

The self-diffusion coefficients, characterizing long-range macroscopic transport of ^7^Li, ^19^F and ^1^H nuclei, are obtained from multinuclear PFG-NMR experiments using ln *I*/*I*_0_ = –*D*^NMR^*γ*^2^(*Δ* – *δ*/3)*δ*^2^*g*^2^,^23^ where *I* and *I*_0_ are the signals in the presence and absence of the gradient respectively, *γ* is the gyromagnetic ratio of the nucleus studied, *Δ* is the interval between the gradient pulses, *δ* is the length of the gradient pulse, and *g* is the magnitude of the gradient pulse. Self-ionic diffusion coefficients in the temperature range 0–60 °C are shown in Fig. 4a.

G_1_–CN–0.1 M LiPF_6_ exhibits much higher ^1^H diffusion coefficients (varying from 1.9 × 10^–12^ to 6.3 × 10^–11^ m^2^ s^–1^ between 0 and 60 °C), nearly one order in magnitude higher compared to G_1_–COOR–0.1 M LiPF_6_ (2.5 × 10^–13^ to 1.8 × 10^–11^ m^2^ s^–1^ between 10 and 60 °C). Following the Stokes–Einstein equation (*D* = *kT*(6π*ηr*_s_)^–1^, where *η*, *D* and *r*_s_ are viscosity, self-diffusion coefficient and effective hydrodynamic (Stokes) radius respectively), the higher viscosity (0.3 Pa s) of G_1_–COOR–0.1 M LiPF_6_ compared to G_1_–CN–0.1 M LiPF_6_ (0.15 Pa s) results in lower ^1^H diffusion coefficients for the ester dendrimer. ^19^F self-diffusion coefficients for both G_1_–CN–0.1 M LiPF_6_ (1.36 × 10^–12^ to 7.8 × 10^–11^ m^2^ s^–1^ between 0 and 60 °C) and G_1_–COOR–0.1 M LiPF_6_ (3.5 × 10^–13^ to 1.1 × 10^–11^ m^2^ s^–1^ between 20 and 60 °C) are found to be in close proximity to their respective ^1^H diffusion coefficient values. The similarities in diffusion coefficient values between ^19^F and ^1^H nuclei for both G_1_–COOR and G_1_–CN signify correlated PF_6_^–^ anion motion with the dendrimer molecules. The lower viscosity of G_1_–CN–0.1 M LiPF_6_ results in nearly one order of magnitude higher ^19^F diffusion coefficient for G_1_–CN–0.1 M LiPF_6_ (1.3 × 10^–11^ m^2^ s^–1^ at 30 °C) compared to that of G_1_–COOR–0.1 M LiPF_6_ (1.1 × 10^–12^ m^2^ s^–1^ at 30 °C). The estimated *R*_PF_6_^–^_ (= *D*_H_/*D*_F_) for ^19^F in G_1_–CN (0.6) and G_1_–COOR (0.8) supports a stronger solvent-correlated PF_6_^–^ motion in G_1_–COOR compared to G_1_–CN. The reason behind this correlation is possibly due to stronger steric hindrance posed by the bulkier *t*-butyl groups to PF_6_^–^ mobility^24^ in G_1_–COOR compared to linear –CN in G_1_–CN. In contrast, lithium-ion diffusion is not at all influenced by the viscosity. Similar values of ^7^Li self-diffusion coefficients are observed in both cases (2.0 × 10^–12^ to 5 × 10^–11^ m^2^ s^–1^ between 30 and 60 °C). The contributions of Li^+^ and PF_6_^–^ towards the total effective conductivity for G_1_–CN, calculated following the Nernst–Einstein equation^23^,25a (*σ*_i_ = *Nq*^2^(*kT*)^–1^*D*_i_, where *σ*_i_ and *D*_i_ are the dc ionic conductivity and diffusion coefficient of the *i*^th^ ion type), are 1.2 × 10^–5^ Ω^–1^ cm^–1^ and 4.9 × 10^–5^ Ω^–1^ cm^–1^ respectively. The higher value of anion conductivity strongly suggests that G_1_–CN is predominantly an anion conductor. On the other hand, the lower contribution of ^19^F (*σ* = 3.9 × 10^–6^ Ω^–1^ cm^–1^ from NMR data) to the total conductivity in G_1_–COOR compared to its ^7^Li diffusion (1.1 × 10^–5^ Ω^–1^ cm^–1^) strongly suggests cation transport in the ester dendrimer. Thus, trapping of anions by the peripheral bulkier ester group in G_1_–COOR makes it a single cationic conductor. Thus, the difference in effective ionic conductivity values between G_1_–CN and G_1_–COOR is mainly due to the difference in the anionic conductivity between them. Manipulation of the chemical constitution of dendrimers *via* variations in the peripheral group, which exert varying degrees of steric hindrance to the mobility of anions, results in a transformation from an anionic to a cationic conductor. This approach is interesting as it becomes the organic analog to the concept of heterogeneous doping introduced by Maier.^26^ Heterogeneous doping has been in the past successfully implemented to account for changes in effective ionic conductivity of solid–solid composites comprised of dispersions of nanometer- to micrometer-sized oxide additives (*e.g.* Al_2_O_3_, SiO_2_) in a weak solid electrolyte, *e.g.* LiI, TlCl_2_.^27^ In this concept, the changes in conductivity have been attributed to the space-charge layer formed at the interface of the weak electrolyte–oxide insulator which directly influences the transition from an anion to a cation conductor as demonstrated in TlCl_2_–Al_2_O_3_. The concept with limited success was later extended to liquids where dispersions of fine oxide particles in liquid electrolytes lead to modest enhancements in the effective conductivity of the liquid.^28^,^29^ To the best of our knowledge the heterogeneous doping concept has not yet been utilized to transform the nature of ion transport in liquids. It is envisaged that the approach presented here is the first of its kind to be adopted in the realm of liquids. This adopted approach is simpler and is expected to be highly efficient and reproducible compared to the addition of oxides, which display considerable non-uniformities in size and chemical functionality of the oxide additive.

In the case of G_1_–CN–0.1 M LiPF_6_, the observed ^7^Li diffusion coefficient is almost an order of magnitude lower compared to its corresponding ^19^F or ^1^H diffusion coefficients, signifying uncorrelated motion of lithium ions with the dendrimer molecules, as expected in the case of weak interactions between –CN and Li^+^. On the other hand, stronger binding between –COOR and Li^+^ in G_1_–COOR–LiPF_6_ leads to close proximity of ^7^Li to the ^1^H diffusion coefficients at all temperatures, signifying a higher correlated motion of lithium ions with the ester molecules. The correlated motion of Li^+^ in G_1_–COOR is further supported by the estimate of the Stokes radius for lithium (*R*_Li_ = *D*_H_/*D*_Li_),25*b* which is equal to 1.4, signifying an almost 1 : 1 co-ordination between G_1_–COOR and Li^+^.

The temperature dependent ionic diffusivity (Fig. 4a) is fitted using the Arrhenius equation and the activation energies for ^7^Li, ^1^H and ^19^F diffusion are tabulated in Table ST4 (ESI†). The observed trend in activation energies for Li^+^ and F^–^ diffusion is as follows: *E*_D(Li)_ (G_1_–CN) (= 0.53 eV) ∼ *E*_D(Li)_ (G_1_–COOR) (= 0.53 eV) and *E*_D(F)_ (G_1_–CN) (= 0.50 eV) < *E*_D(F)_ (G_1_–COOR) (= 0.70 eV). This trend further suggests that the viscosity mainly influences the activation energy of ^19^F diffusion and not ^7^Li diffusion. Similarly, the Li^+^ diffusion activation energy (*E*_D(Li)_) between G_1_–CN and G_1_–COOR leads to similar activation energy of conductivity, as obtained from ac impedance spectroscopy (Fig. 2a). Hence, the underlying mechanism of lithium conduction is similar to the lithium diffusion mechanism, and the difference in the effective conductivity is mainly determined by the differences in anion mobility. The temperature dependent viscosity (Fig. 2b) and diffusion (Fig. 4a) do not show a clear VTF-like behaviour (*i.e.* curvature-like profile). So, employing the VTF analysis will not be appropriate for analyzing and correlating the diffusion, viscosity and conductivity data together. Additionally, first-generation dendrimers are considered as viscous liquids with considerably simpler molecular architectures than the higher generation dendrimers (G_*n*_, *n* > 2) or polymers. So, based on these aspects we considered thermally activated diffusion, viscosity and conductivity (*i.e.* Arrhenius), rather than segmental motion-driven ion transport where VTF fitting would be more appropriate.

The transference numbers of ^7^Li(*t*_+_) and ^19^F(*t*_–_) are calculated from ionic diffusion coefficients following the equation: *t*_+_ = (1 – *t*_–_) = *D*_+_(*D*_+_ + *D*_–_)^–1^ where *D*_+_ and *D*_–_ are the cationic and anionic diffusion coefficients respectively. Fig. 4b shows the temperature dependent cationic (Li^+^) and anionic (PF_6_^–^) transference numbers for both G_1_–COOR–0.1 M LiPF_6_ and G_1_–CN–0.1 M LiPF_6_. G_1_–COOR–0.1 M LiPF_6_ exhibits an extremely high lithium transference number (*t*_+_) of 0.9 at all experimental temperatures, whereas the Li^+^ transference number of G_1_–CN–0.1 M LiPF_6_ is observed to be as low as 0.2, almost comparable to conventional PEO-based polymer electrolytes. Lower values of ^19^F diffusion coefficient compared to Li^+^ diffusion coefficient in the G_1_–COOR electrolyte lead to an extremely high Li^+^ transference number, suggesting predominantly a cationic conductor. In comparison, G_1_–CN presents the opposite scenario, where a very high anionic transference number (*t*_–_ = 0.8) is observed compared to lithium, implying favorable anion transport. As discussed earlier, the viscosity and steric hindrance of bulky peripheral –COOR groups affect the mobility of larger anions, and this trapping effect results in the extremely high Li^+^ transference number in G_1_–COOR, in spite of the lower conductivity compared to the G_1_–CN electrolyte. A significant difference in cationic transference number between G_1_–CN (*t*_+_ = 0.2) and G_1_–COOR (*t*_+_ = 0.9) further suggests that the present approach is highly effective in manipulating the nature of ion transport in dendrimer electrolytes. We attempted the estimation of the Li^+^ ion transference number using the electrochemical method proposed by Evans, Vincent and Bruce.20a,^30^ This method has been predominantly employed to estimate the cation transference numbers of liquid and polymer electrolytes. Molecular solvent-based liquid and polymer electrolytes exhibit both cation and anion conductivity in one system. The transference number of one ion type is usually greater than the other; however, both are appreciably high and contributions to conductivity from the minority carrier cannot be neglected. So, the G_1_ dendrimer electrolytes do not exactly match the criteria for applicability of this method. The *t*_+_ for G_1_–CN–0.1 M LiPF_6_ is estimated to be ≈0.4, which was higher than our estimates from NMR (*t*_+_ = 0.2). Thus, both electrochemical and NMR measurements conclude that the G_1_–CN is an anion conductor. On the other hand, the estimated *t*_+_ of G_1_–COOR–LiPF_6_ was 0.3, instead of 0.9 as predicted from the diffusion NMR measurements. The reason for the large discrepancy between the values in G_1_–COOR is due to a combination of various factors. Higher viscosity leads to slower anion kinetics during polarization. An extremely slow anion diffusion coefficient also leads to uncertainties in maintaining the necessary condition of zero anion flux in the steady state. At this juncture, the possibility of an imminent application of the novel G_1_ dendrimer electrolyte in an electrochemical device is remote and non-trivial. However, it is strongly envisaged that the present dendrimer and similar systems will have strong implications in various applications such as rechargeable batteries, sensors and actuators. The present dendrimers exhibit very high anion and cation transference numbers, with the conductivities being on a par with those of many polymer-based single ion conductors.10a,^19^ The remarkably high cation transference number (*t*_+_ = 0.9) of G_1_–COOR–LiPF_6_ prompted us to perform electrochemical characterizations for potential application as an electrolyte or as a co-solvent in rechargeable batteries. We discuss here some of the studies which may trigger electrolyte designs based on dendrimers, in general polymeric systems for rechargeable batteries based on lithium. The cell configurations and electrode assemblies employed for the studies are exactly similar to those used for molecular based solvent electrolytes.

**Fig. 5:**
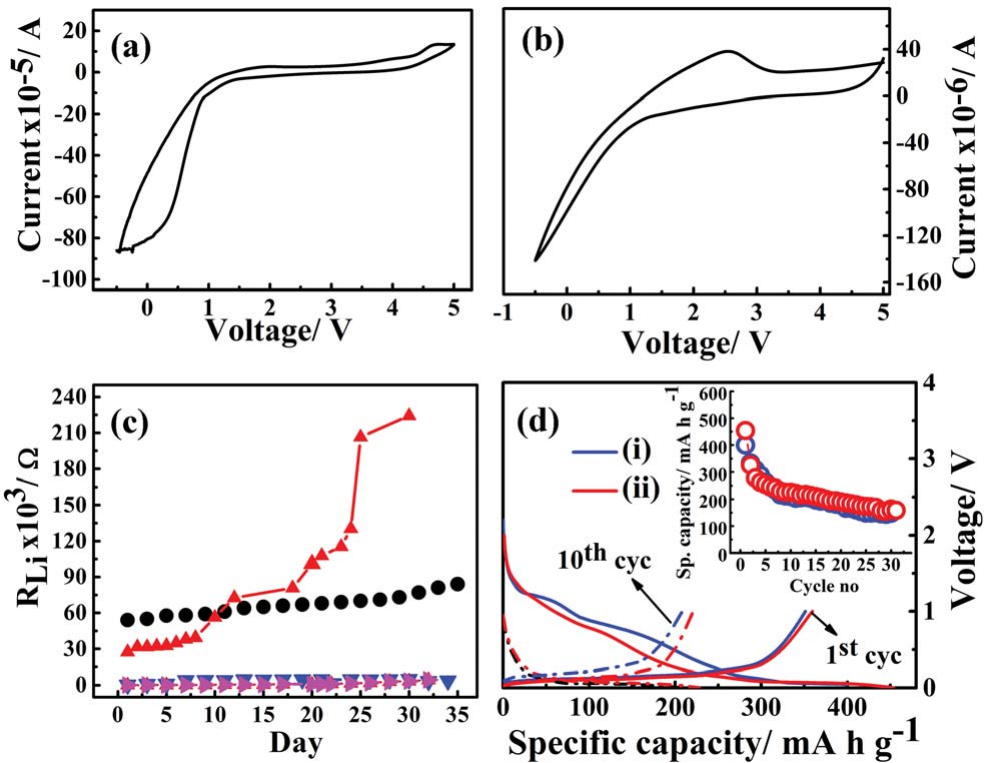
Cyclic voltammograms of G_1_–CN–0.1 M LiPF_6_ (a) and G_1_–COOR–0.1 M LiPF_6_ (b) with stainless steel as working and lithium as reference and counter electrodes. (c) Lithium interface stability of G_1_–CN (black circles), G_1_–COOR (red triangles), 50% G_1_–CN–(EC–DMC) (pink triangles), 50% G_1_–COOR–(EC–DMC) (blue triangles) dendrimer electrolytes. (d) Battery cycling performance of ternary 50% G_1_–CN–(EC–DMC–LiPF_6_) (i) (blue) and 50% G_1_–COOR–(EC–DMC–LiPF_6_) (ii) (red) electrolytes with Li/electrolyte/graphite cell configuration at *C*/10 constant current rate. Inset shows specific capacity (mA h g^–1^) *vs.* cycle number for both ternary electrolytes.

The electrochemical potential windows of G_1_–CN–LiPF_6_ and G_1_–COOR–LiPF_6_ electrolytes were studied *via* cyclic voltammetry with Li|G_1_-dendrimer–LiPF_6_|SS (stainless steel) cell configuration at a scan rate of 1 mV s^–1^, and the results are represented in Fig. 5a and b respectively. The cyclic voltammograms clearly shows that the G_1_–CN system does not support stable reversible cycling of Li, whereas G_1_–COOR shows a lithium stripping peak (at ∼2 V) followed by reductive deposition too. The higher cathodic (deposition) currents observed for G_1_–CN as compared to G_1_–COOR are consistent with the higher conductivity of G_1_–CN compared to G_1_–COOR. Improved reversibility in lithium cycling for G_1_–COOR compared to G_1_–CN is a consequence of the higher lithium transference number and faster ionic diffusion at the lithium electrode. Both of these dendrimers show a stable electrochemical window of 4 V as observed from cyclic voltammetry.

The electrochemical stability of G_1_–COOR–LiPF_6_ and G_1_–CN–LiPF_6_ electrolytes at lithium metal interfaces were investigated over a period of 35 days by ac impedance spectroscopy in a symmetrical Li|G_1_-dendrimer|Li cell configuration, as shown in Fig. 5c. The lithium interface resistance (*R*_Li_) was evaluated from the Nyquist plots as shown in Fig. S4.† The lithium interfacial resistance (*R*_Li_) of G_1_–COOR–0.1 M LiPF_6_ on the first day (5.4 × 10^4^ Ω) is higher than that of G_1_–CN–0.1 M LiPF_6_ (2.7 × 10^4^ Ω). However, a sudden increase in interfacial resistance is observed in the case of G_1_–CN–LiPF_6_ after the 15^th^ day. At the 30^th^ day, *R*_Li_ of G_1_–CN–0.1 M LiPF_6_ increased to 2.2 × 10^5^ Ω (10 times increase in magnitude compared to day 1). On the other hand, the G_1_–COOR electrolyte displayed a marginal increase over the same period reaching the value of 7.7 × 10^4^ Ω on the 30^th^ day (1.3 times increase in magnitude). This result indicates a slower rate of growth of the passivation layer at the lithium interface for G_1_–COOR–0.1 M LiPF_6_ compared to G_1_–CN–0.1 M LiPF_6_. The improved stability can be directly attributed to the high *t*_Li^+^_ in G_1_–COOR–0.1 M LiPF_6_ which improves the charge transfer kinetics at the electrode|electrolyte interface. Following this, galvanostatic charge/discharge cycling measurements were performed (rate = *C*/10). The pristine dendrimer–salt system *i.e.* G_1_–COOR–LiPF_6_ (and G_1_–CN–LiPF_6_) exhibited poor galvanostatic cycling. The capacity faded to very low values within a few cycles. We attribute the failure to the high viscosity of the pristine dendrimer–salt system which resulted in poor charge kinetics at the electrode|electrolyte interface.

Following the unsatisfactory battery cycling of the pristine dendrimer electrolytes, the cells were assembled with a mixture (by volume) containing 50% ethylene carbonate (EC)–dimethyl carbonate (DMC) (EC : DMC = 1 : 1 by v/v) and 50% of G_1_–COOR (G_1_–CN) and LiPF_6_. G_1_–COOR–EC–DMC–LiPF_6_ (as well as G_1_–COOR–EC–DMC–LiPF_6_) exhibited a voltage stability of 3 V with stainless steel (as working electrode) and lithium foil (Aldrich) as the counter and reference electrodes (Fig. S5†). The lithium metal is passivated even better in the case of G_1_–COOR–EC–DMC (or G_1_–CN–EC–DMC) compared to the pristine dendrimers. No significant change in the interface resistance is observed in the case of the ternary mixture G_1_–COOR–EC–DMC (and G_1_–CN–EC–DMC) (Fig. 5c). This strongly suggests that the G_1_ dendrimers with further chemical design modifications (leading to lower viscosity) can be employed as both alternative electrolytes and electrolyte additives in conventional liquid electrolytes. With regard to the latter issue, there have been a few interesting reports on boron-based additives^31^ aimed at stabilizing both the cathode/anode|electrolyte interfaces. While the boron-based additives aid in electron transport, the present dendrimers aid in ion conductivity. Hence, to the best of our knowledge the PETIM dendrimers are the first of their kind where the additive stabilizes the electrode|electrolyte interface *via* promotion of ion transport.

Fig. 5d represents the galvanostatic cycling performance of G_1_–CN–EC–DMC–LiPF_6_ and 50% G_1_–COOR–EC–DMC–LiPF_6_ with graphite as working electrode and lithium metal as reference and counter electrodes, respectively. The charge and discharge cycling were done at a constant current rate of *C*/10 over a 0–2.5 V voltage range for the dendrimers. In Fig. 5b, the first discharge curve shows two distinct reductive plateaux in the ranges 0.5–0.8 V and 0.9–1.5 V corresponding to reductive degradation of EC solvent (SEI formation) *via* single and double reduction processes,^32^ which vanish on further cycling in the lithium insertion process. The broad plateau at 0.5–0.8 V signifies decomposition of the G_1_–CN molecule at the graphite surface. The charge plateau appears at 0.18 V corresponding to lithium de-insertion processes. The appearance of reductive and oxidative peaks agrees with the cyclic voltammetry results (ESI Fig. S6†). G_1_–CN–EC–DMC–LiPF_6_ shows a 1^st^ discharge capacity of 400 mA h g^–1^ which decreases to 330 mA h g^–1^ in the 2^nd^ cycle. In the 30^th^ cycle, the capacity stabilized at 150 mA h g^–1^. The 1^st^ charge capacity is equal to 354 mA h g^–1^ and this stabilized to 148 mA h g^–1^ in the 30^th^ cycle. Coulombic efficiency increases from 88% (1^st^ cycle) to 89% (5^th^ cycle) and stabilized at 99% in the 30^th^ cycle. The low coulombic efficiency in the 1^st^ cycle is a consequence of irreversible capacity loss during the formation of the SEI film, which stabilizes over successive cycling leading to higher coulombic efficiency over successive cycles. Similar cycling behavior for ternary G_1_–COOR–EC–DMC–LiPF_6_ is observed except that the additional plateau at 0.9–1.5 V is absent in this case. The disappearance of this reductive plateau at 0.9–1.5 V indicates lesser decomposition of EC (suppression of the two-electron transfer process of EC)^32^ and better stability of the G_1_–COOR dendrimer at the graphite electrode surface (clear from cyclic voltammetry in Fig. S5†). The G_1_–COOR–EC–DMC–LiPF_6_ specific capacity in the 1^st^ discharge cycle is 453 mA h g^–1^ which decreases to 325 mA h g^–1^ in the 2^nd^ cycle. In the 30^th^ cycle, the capacity stabilized at 160 mA h g^–1^. The 1^st^ charge capacity is 359 mA h g^–1^ and stabilized at 153 mA h g^–1^ in the 30^th^ cycle. Coulombic efficiency increases from 79% (1^st^ cycle) to 85% (5^th^ cycle) and eventually stabilized at 96% in the 30^th^ cycle. Thus, the ternary dendrimer electrolyte can be successfully cycled with a graphite electrode and may hold promise in lithium battery applications.

## Conclusion

In conclusion, we have demonstrated here a novel dendrimer–salt based ion conductor with high ion transference for prospective applications as an electrolyte in diverse devices such as rechargeable batteries, sensors and actuators. The transference number achieved here is the highest reported so far in dendrimers and polymer electrolytes. We have comprehensively demonstrated for the first time that ion conductivity and transference number can be manipulated by varying the chemical nature of the dendrimer peripheral group. The chemical nature of the peripheral group completely determines the solvation, *i.e.* quantum of free charge carriers, the mobility of the free charge carriers and the electrochemical properties. It is interesting to note that changes in peripheral chemical functional groups which affect the viscosity of the solution do not at all influence the cation diffusivity. We anticipate similar observations for other alkali ions provided the anion remains the same or bulkier than the PF_6_^–^ anion. In the case of other monovalent cations (K^+^, Rb^+^, Cs^+^), factors specific to the metal ions need to be considered to achieve similar trends in ion transport in dendrimer electrolytes. Given the advancements in polymer processing, it is strongly proposed that similar strategies as discussed here can also be adopted in designing novel solid polymers with a variety of metal salt single-ion conducting polymer electrolytes.

## Supplementary Material

Supplementary information
