# Supplementary material for: A single cation or anion dendrimer-based liquid electrolyte
Source: Chem Sci. 2016 Jan 29;7(5):3390–8. doi: 10.1039/c5sc04584c (PMC6007127; doi:10.1039/c5sc04584c)
Supplement: Supplementary file 1 [file SC-007-C5SC04584C-s001.pdf]

Electronic Supplementary Material (ESI) for Chemical Science.
This journal is © The Royal Society of Chemistry 2016

Supporting Information

A single cation or anion dendrimer-based liquid electrolyte

Sudeshna Sen, Rudresha B. Jayappa, Haijin Zhu, Maria Forsyth, Aninda J. Bhattacharyya
Solid State and Structural Chemistry Unit, Indian Institute and Science, Bangalore, 560012, India.
Institute for Frontier Materials, deakin University, Waurn Ponds, VIC3216, Australia

Experimental Section: Materials and Methods

All chemicals are of analytical grade and used as received without further purification.

Synthesis of dendrimers: The G_1-PETIM with different peripheral end groups are synthesized from bis nitrile (G_0-CN) via an iterative reaction sequence following an earlier report.[1] In brief, the first step of iterative sequence consists of synthesis of bis nitrile (G_0-CN) from acetonitrile. Aqueous NaOH is added to acetonitrile, purified by passing through neutral alumina chamber, followed by vigorous stirring at room temperature (25 ^{o}C). First generation cyano terminated PETIM dendrimer (G_1-CN) is synthesized from bis nitrile through alternative Michael addition and reduction reactions as described in previous report.[15] Nitrile group of G_0-CN is hydrogenated by Raney-Co and H_2 (50 bar, 70 ^{o}C) in water solvent to obtain amine terminated G_0-NH_2, which is used as monomer to synthesize first generation-COOR (R= ^{t}Butyl), -COOH and -OH terminated PETIM dendrimers.[2] Michael addition of tert-butyl acrylate to G_0-NH_2 leads to tert-butyl ester terminated dendrimer (G_1-COOR, R= ^{t}Butyl), followed by reduction of ester with $LiAlH_4$ in THF to yield alcohol terminated G_1-OH. Ester hydrolysis in presence of acetyl chloride leads to formation of carboxylic acid (-COOH) terminated PETIM dendrimer (G_1-COOH). The series of dendrimers with different functional groups are characterized by Fourier transform infra-red spectroscopy (FTIR), ^{1}H and ^{13}C nuclear magnetic resonance spectroscopy (NMR).[1,2] The representative FTIR and ^{1}H NMR characterization of G_1-COOR are provided in Fig. S1 and S2.

Sample preparation for ionic conductivity measurements: Requisite amount of lithium trifluorophosphate ($LiPF_6$) is dissolved in the pristine dendrimers to obtain a concentration range of 0.05-0.2 M. The ionic conductivity is estimated using ac-impedance spectroscopy (Novocontrol Alpha-A; frequency range: 1 to 1×10^6 Hz). The electrolyte is sandwiched between two stainless steel electrodes in home-built glass cells for conductivity measurements. Cell constant for all the measurements is maintained 0.05 cm^{-1}. The conductivity cells are assembled in home-built glass jackets for temperature dependent conductivity measurement. The glass jacket with the conductivity cell is inserted into thermostat (FP50MC) containing ethylene glycol-water mixture to measure temperature dependent data. All measurements are performed within the temperature ranges (0-60) ^oC at a temperature interval of 5 ^oC for both heating and cooling cycles. All of the sample preparations and cell assemblies are carried out in argon filled glove box (MBraun, MB 20G LMF, pressure: 3 mbar, H_2O < 0.5 ppm, O_2< 0.5 ppm)

Structural characterization: Viscosities at various temperatures are obtained from steady state rheology, evaluated from shear independent region of viscosity versus shear rate plot. The rheological measurements are performed on AR-G2 stress-controlled rheometer (TA Instruments). The device is equipped with an ETC (environmental test chamber) suitable for studying polymer melts under N_2 atmosphere. Parallel-plate geometry with 25 mm plate diameter and 1 mm gap distance is used for the measurements. The phase separation process could be followed *in situ* by small amplitude oscillatory measurements (for e.g. 1% strain). Successive frequency sweeps (in the range of 100-0.01 rad/s) are performed during experiment. Fourier transform infrared (FTIR) spectra at various $LiPF_6$ salt concentrations and at different temperatures (RT to 70 ^oC with 10 ^oC interval) are recorded on a Perkin Elmer Spectrum 2000Spectrometer at a spectral resolution of 4 cm^{-1} in the transmission

mode. The PFG-NMR diffusion experiments are carried out on a Bruker Advance III 300 MHz wide bore spectrometer (with proton Larmor frequency of 300.13 MHz) equipped with a 5 mm diff50 probe. The pulse-field gradient stimulated echo (PFG-STE) pulse sequence is used to obtain diffusion coefficients. The maximum gradient strength is 29.454 T/m. In the present study, the interval between the gradient pulses (Δ) is varied from 5 to 10 ms, length of gradient pulse (δ) is set between 1 and 4 ms, and g is optimized to a suitable strength range from 0.3 to 29.4 T/m according to the diffusion coefficients. Recycle delays between are set to 5 s for all the diffusion experiments. The sample temperatures in the probe for the variable temperature experiments are calibrated by using the relative chemical shift separation between the OH resonance and CH_3 resonance of dry methanol.[3] The sample temperatures for variable temperature experiments are calibrated with lead nitrate, using the method described in literature [4].

Electrochemical measurements : Galvanostatic Cycling: Galvanostatic cycling is performed using an Arbin Instruments (BT 2000 Corp., USA) at C/10 C-rate in the voltage range of (0-2.5) V (versus Li^+/Li). The electrochemical characterizations are carried out using Swagelok^{TM} cells with lithium foil (Aldrich) as the counter and reference electrodes, graphite as the working electrode, Whatman glass fiber as separator and pristine dendrimer-salt system i.e. G_1-COOR-$LiPF_6$ (and G_1-CN-$LiPF_6$) as electrolyte. For active electrode material i.e. graphite is mixed with PVDF in a weight ratio of 9:1 (graphite: PVDF) and slurry is made with N-methyl-2-pyrrolidone (NMP) solvent. As prepared slurry is cast on circular Al foil (thickness = 20 μm, Ranga Techno Impex) and dried under vacuum at 110 °C overnight.

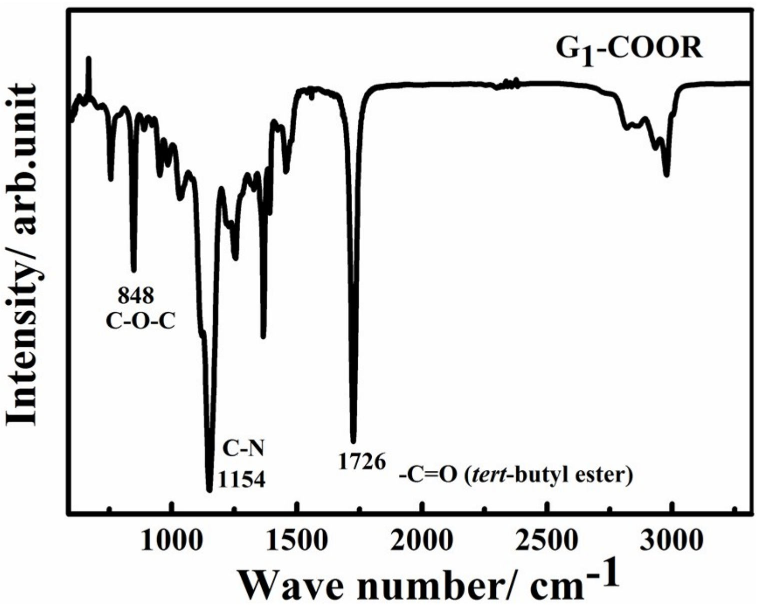

Fig. S1 FTIR spectra of G_1-COOR dendrimer.

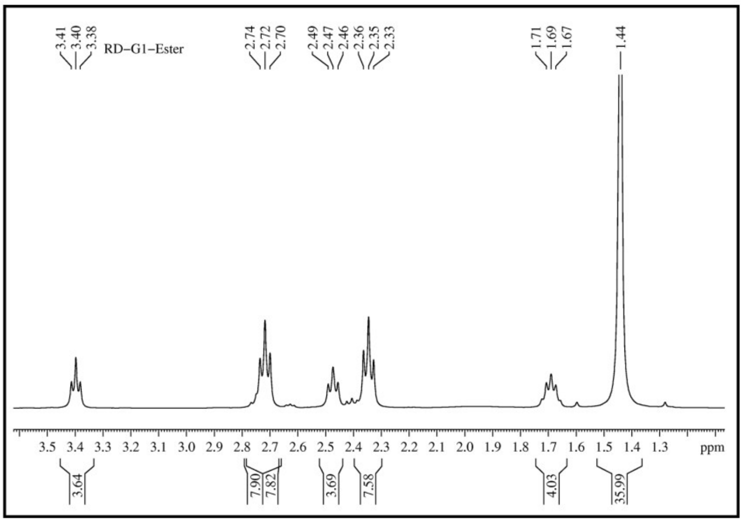

Fig. S2 ^{1}H NMR spectra of G_1-COORdendrimer.

Table ST1: Room temperature conductivity of G_1-CN-x M $LiPF_6$.

x/ M	RT conductivity/ $\Omega^{-1}cm^{-1} \times 10^5$
0.05	0.78
0.1	1.9
0.2	1.2

Table ST2 Activation energies from Arrhenius fitting of viscosity and conductivity data.

Sample	Conductivity ($\times 10^6$)/Ω^{-1}cm^{-1} at 25° C	Viscosities (Pa.s) at 30° C	E_a/ eV	A/ Ω^{-1}cm^{-1} $\times 10^5$	E_η / eV	A_η / Pa.s
G_1-CN-0.1M $LiPF_6$	19.14	0.15	0.54	3.6	0.19	$1x\ 10^{-3}$
G_1-COOR-0.1M $LiPF_6$	1.96	0.33	0.58	9.8	0.54	$3.16\ x\ 10^{-10}$
G_1-OH-0.1M $LiPF_6$	0.90	4.5	0.58	22.4	0.51	$1.2\ x\ 10^{-8}$
G_1-COOH-0.1M $LiPF_6$	0.98	6.8	0.58	19.6	0.45	$1.5\ x\ 10^{-7}$

Table ST3 VTF fitting parameters of σ vs 1000/T plot (Fig. 2a).

$$\sigma = \sigma_0 \exp\left[\frac{-B}{R(T-T_0)}\right]$$

sample	B/R(K)	T0 (K)	σ0 (scm^{-1})
G_1-CN-0.1M $LiPF_6$	743	201	0.03
G_1-est-0.1M $liPF_6$	807	196	0.003
G_1-OH-0.1M $liPF_6$	944	193	0.0069
G_1-COOH-0.1M $liPF_6$	808	196	0.0015

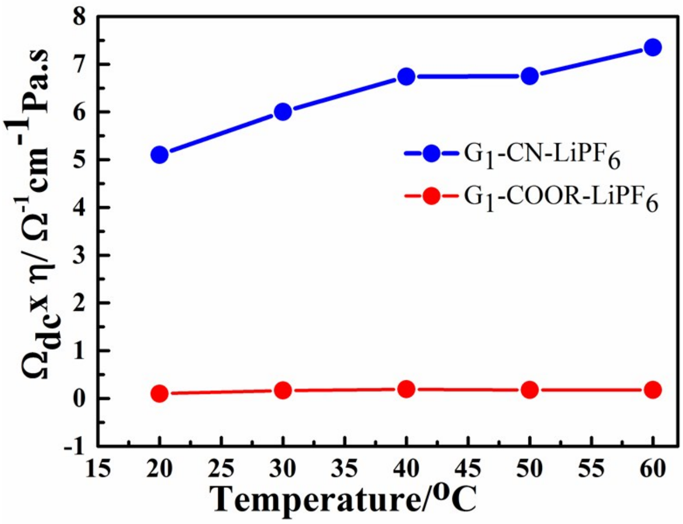

Fig. S3 Validation of Stokes equation for G_1-CN-0.1M $LiPF_6$ (blue circle) and G_1COOR-0.1M $LiPF_6$ (red circle)

Stokes equation,

$$\sigma_{dc} = \frac{Nq^2}{6\pi\eta r_s} \text{......Equation SE1}$$

$$\sigma_{dc} \times \eta = \frac{Nq^2}{6\pi r_s} \text{............Equation SE2}$$

$\sigma_{dc} \times \eta$ = invariable with temperature for full dissociation of salt in electrolyte.

Table ST4: Activation energies from Arrhenius fitting of self-diffusion coefficients

Samples	$E_D(Li^+)$ eV	$E_D(F^-)$ eV	$E_D(^1H)$ eV
G_1-CN-0.1M $LiPF_6$	0.53	0.50	0.46
G_1-COOR-0.1M $LiPF_6$	0.53	0.70	0.70

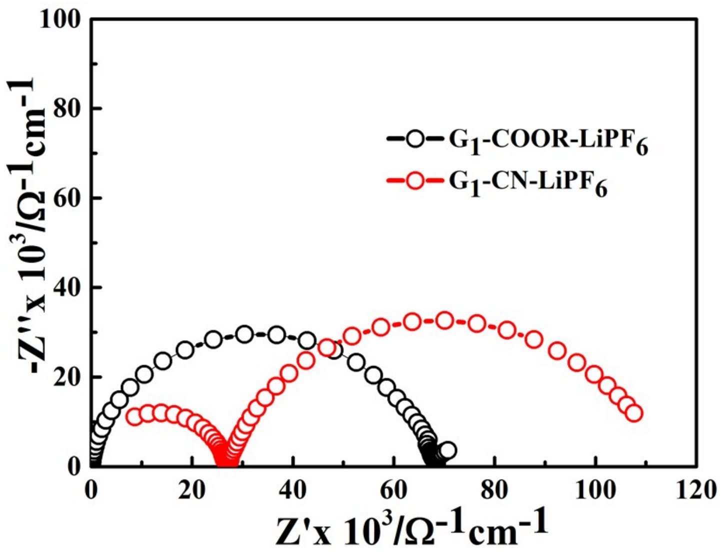

Fig. S4 Nuiquist plot of Li/dendrimer/Li symmetrical cell for G_1-COOR-$LiPF_6$ (black) and G_1-CN-$LiPF_6$(red) at 21st day.

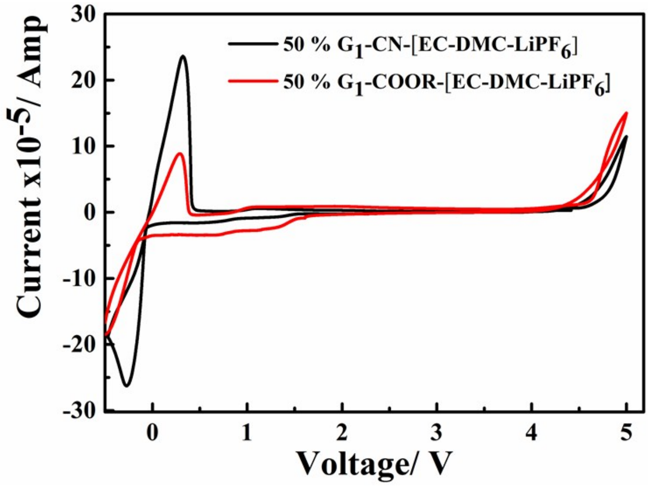

Fig. S5: Cyclic Voltammograms of ternary Dendrimer-[EC-DMC-$LiPF_6$] with stainless steel as working electrode and lithium as reference and counter electrode.

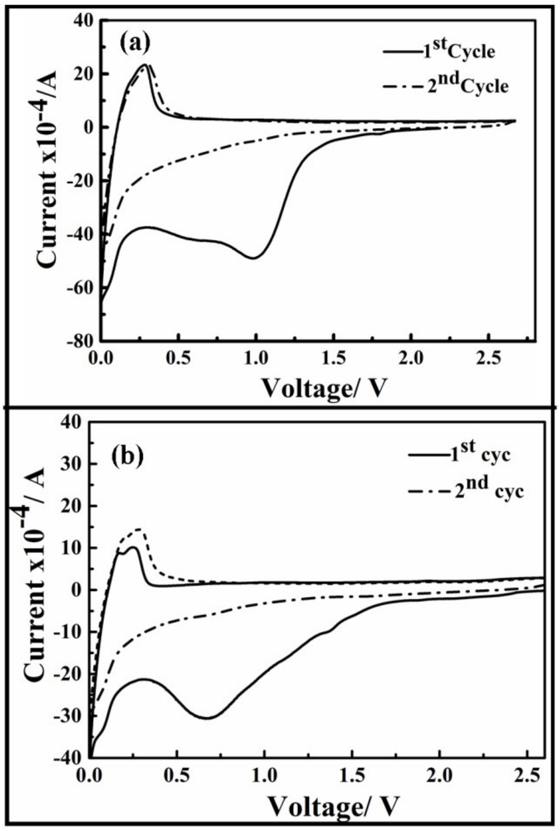

Fig. S6: Cyclic Voltammograms of ternary G_1-CN-EC-DMC-$LiPF_6$(a) and G_1-COOR-EC-DMC-$LiPF_6$(b) electrolyte with graphite as working electrode and lithium as reference and counter electrode at scan rate 0.2mVs^{-1}.

References (ESI) :

1. a) T. R. Krishna, N. Jayaraman, *J. Org. Chem.* **2003,** *68*:9694. b) G. Jayamurugan, N. Jayaraman, *Tetrahedron,* 2006, 62:9582.

2. a) S. Jain, A. Kaur, R. Puri, P. Utreja, A. Jain, M. Bhide, R. Ratnam, V. Singh, A. S.s Patil, N. Jayaraman, G. Kaushik, S. Yadav, K. L. Khanduja, *Eur. J. Med. Chem.,* 2010, **45**, 4997-5005. b) T. Rama Krishna, Jain, U. S. Tatub, N. Jayaramana, *Tetrahedron,* 2005, **6***,* 4281–4288.

3. C. Ammann, P. Meier, A. E. Merbach *J. Mag. Res.,* 1982, **46**, 319.

4. a) A. V. Geet, Anal. Chem. 1970, **42**, 679. b) A. Bielecki, D. P.Burum, *J. Magn. Reson*. Ser. A, 1995, **116**, 215.
